# Ethylene Activates the *EIN2*-*EIN3*/EIL1 Signaling Pathway in Tapetum and Disturbs Anther Development in *Arabidopsis*

**DOI:** 10.3390/cells11193177

**Published:** 2022-10-10

**Authors:** Ben-Shun Zhu, Ying-Xiu Zhu, Yan-Fei Zhang, Xiang Zhong, Keng-Yu Pan, Yu Jiang, Chi-Kuang Wen, Zhong-Nan Yang, Xiaozhen Yao

**Affiliations:** 1 Shanghai Key Laboratory of Plant Molecular Sciences, College of Life Sciences, Shanghai Normal University, Shanghai 200234, China; 2 Development Center of Plant Germplasm Resources, College of Life Sciences, Shanghai Normal University, Shanghai 200234, China; 3 National Key Laboratory of Plant Molecular Genetics, CAS Center for Excellence in Molecular Plant Sciences, Institute of Plant Physiology and Ecology, Chinese Academy of Sciences, Shanghai 200032, China

**Keywords:** ethylene, anther wall, tapetum, microspore

## Abstract

Ethylene was previously reported to repress stamen development in both cucumber and *Arabidopsis*. Here, we performed a detailed analysis of the effect of ethylene on anther development. After ethylene treatment, stamens but not pistils display obvious developmental defects which lead to sterility. Both tapetum and microspores (or microsporocytes) degenerated after ethylene treatment. In *ein2-1* and *ein3-1 eil1-1* mutants, ethylene treatment did not affect their fertility, indicating the effects of ethylene on anther development are mediated by *EIN2* and *EIN3*/EIL1 in vivo. The transcription of *EIN2* and *EIN3* are activated by ethylene in the tapetum layer. However, ectopic expression of *EIN3* in tapetum did not induce significant anther defects, implying that the expression of *EIN3* are regulated post transcriptional level. Consistently, ethylene treatment induced the accumulation of *EIN3* in the tapetal cells. Thus, ethylene not only activates the transcription of *EIN2* and *EIN3*, but also stabilizes of *EIN3* in the tapetum to disturb its development. The expression of several ethylene related genes was significantly increased, and the expression of the five key transcription factors required for tapetum development was decreased after ethylene treatment. Our results thus point out that ethylene inhibits anther development through the *EIN2*-*EIN3*/EIL1 signaling pathway. The activation of this signaling pathway in anther wall, especially in the tapetum, induces the degeneration of the tapetum and leads to pollen abortion.

## 1. Introduction

Ethylene (C_2_H_4_) is a simple but important gaseous phytohormone. It regulates various developmental processes, including seed germination, leaf development, senescence, sex determination, fruit ripening, and abiotic and biotic stress response [1]. Ethylene is produced from methionine (Met), and the biosynthetic pathway involves three enzymatic steps. Met is firstly converted to S-adenosyl-L-methionine (SAM) by SAM-synthetases, then converted to 1-aminocyclopropane-1-carboxylic acid (ACC) by ACC synthases (ACS). As an immediate ethylene precursor, ACC is converted to ethylene by ACC oxidases (ACO) [2]. In *Arabidopsis*, the ethylene receptor family members, [ETHYLENE RESPONSE SENSOR 1 (ERS1), ERS2, ETHYLENE RESPONSE 1 (ETR1), ETR2, and ETHYLENE-INSENSITIVE 4 (EIN4)], activate CONSTITUTIVE TRIPLE RESPONSE 1 (CTR1) in the absence of ethylene [3]. CTR1 is a serine/threonine protein kinase. It acts as a negative regulator of the ethylene signaling pathway by inhibiting the *EIN2* protein [4]. In the presence of ethylene, the inactivation of CTR1 releases the repression of *EIN2*. *EIN2* plays an essential role in the stabilizing of *EIN3* and *EIN3*-LIKE 1 (EIL1) proteins which are key transcriptional factors regulating the downstream ethylene response genes such as ETHYLENE RESPONSE FACTORs (ERFs) [5].

During plant reproduction, ethylene and its signaling pathway are involved in several developmental processes. In *Arabidopsis*, ethylene signaling components such as CTR1, *EIN2*, *EIN3*, and EIL1 are required for the degeneration of synergid cells in the embryo sac to ensure successful fertilization and prevent supernumerary pollen tubes [6,7,8]. Cucumber (*Cucumis sativus*) is a monoecious plant with unisexual flowers, and has been used as a model plant for plant sex determination and differentiation. In the cucumber female flower, anther development is arrested as soon as the differentiation of anther and filament in stamen primordium [9,10]. It has been well documented that exogenous ethylene application promotes monoecious cucumber to produce female flowers [11,12,13]. The *A*, *F* and *M* loci that genetically control the sex determination especially governs the development of female flowers in cucumber have been cloned and showed to encode three ACC synthases, *CsACS11* (A), *CsACS1G* (F) and *CsACS2* (M) [14,15,16]. All of these results indicate the function of ethylene in cucumber for the formation of the female flower. Some evidences from cucumber and *Arabidopsis* have proven that the ethylene-induced female flowers is a result of stamen development inhibition. For example, overexpression of the cucumber ethylene synthesis gene (*CsACO2*) or the down-regulation of the ethylene receptor *CsETR1* in *Arabidopsis* resulted in abnormal stamen formation [17,18]. In *Arabidopsis*, the double loss-of-function mutation of *ERS1* and *ETR1* leads to both male and female gametophyte development defects in an *EIN2*-dependent manner [19]. During female flower development, expression of *CsETR1* was decreased and anther-specific DNA damage became detectable [9,18]. It has been proposed that the ethylene-inducible calcium dependent nuclease *CsCaN* may be responsible for the DNA damage of anther primodia in cucumber female flower [20].

It is quite clear that ethylene represses stamen development in both cucumber and *Arabidopsis*, while the exact mechanisms and the detailed roles of ethylene during anther and pollen development remain elusive. In *Arabidopsis*, the anther wall contains four somatic cell layers: the epidermis, endothecium, middle layer, and tapetum [21]. They form an anther locule (anther theca), where the diploid microsporocytes undergo meiosis and two rounds of pollen mitosis to develop into mature pollen grains. Here, we investigate the effect of ethylene in each cell layers of anther wall and microspores and how the ethylene signaling pathway acts during this process. We found that ethylene treatment leads to shortened filaments and severe degeneration for tapetum and microspores. Our results pointed out that ethylene treatment induces the *EIN2*-*EIN3*/EIL1 signaling pathway in the tapetum, then repressing the growth in anthers.

## 2. Materials and Methods

### 2.1. Plant Material, Growth Conditions and Mutant Identification

The *Arabidopsis* ecotype used in this study is the Columbia-0. *ein2-1*, *ein3-1*, *eil1-1*, *ein3-1 eil1-1*, *etr1-7 ers1-2* mutants have been described previously [19,22,23,24]; these mutants seeds were kindly provided by Chi-Kuang Wen (Chinese Academy of Sciences, Shanghai 200032, China). *can1-1* (GABI_022B01) and *can2-1* (SALK_023120.55.75.n) were obtained from Arabidopsis Biological Resource Center (www.arabidopsis.org (accessed on 10 February 2015 and 15 July 2015)). *can1-1 can2-1* was produced in this study by genetic crosses. *ein2-1* and *ein3-1* are point mutants identified by enzyme digestion after PCR [22,23]. *etr1-7* also has a single nucleotide substitution, which resulted in a stop codon at Trp-74 [3]. *eil1-1, ers1-2, can1-1* and *can2-1* are T-DNA insertion mutants, and the identification of *eil1-1* and *ers1-2* mutant was also described [23,24]. Primers for the identification of all these mutants are listed in Supplementary Table S1. All plant materials were grown at ~22 °C under 16 h light/8 h dark photoperiod in a growth room.

### 2.2. Ethylene Gas Treatment

The growing inflorescences (with about three just opened buds) of 28-days-old plants were marked with red lines. Arabidopsis plants were placed in an airtight chamber (30 cm × 20 cm × 20 cm, the volume is 12 L), and ethylene gas was injected into the chamber (100 µL L^−1^). Another chamber injected with the same volume of air was used as a control. The chambers were then put in a growth room for a certain time (6 h, 12 h, or 24 h). To treat mutants such as *ein2-1*, *ein3-1*, *eil1-1*, *ein3-1 eil1-1*, *can1-1, can2-1* and *can1-1 can2-1*, a pot of wild-type plants (Col-0) was placed in the same chamber to confirm an effective ethylene treatment.

### 2.3. Microscopy

Plants were imaged using a Nikon digital camera (D-7000). Alexander staining was performed as described [25]. In brief, the anthers at later stage 12 or stage 13 were dissected under a dissecting microscope and immersed in an Alexander staining solution for about 1 h before the images were taken. For the production of semi-thin sections, flower buds were fixed by formalin-acetic acid (FAA) for 3 days and then embedded in Spurr’s epoxy resin and cut into 1-μm thick sections, stained with toluidine blue, then photographed with an Olympus BX51 digital camera [26]. Fluorescence detection involving laser scanning confocal microscopy (LSCM) for transgenic plants expressing p*EIN2*:*VENUS*, p*EIN3*:*VENUS*, or p*EIN3*:*EIN3*-*GFP* involved a Zeiss LSM510.

### 2.4. Molecular Cloning

The *VENUS* was amplified and inserted into the XbaI-SalI site to modify vector pCAMBIA1300 (purchased from the CAMBIA center) into pCAMBIA1300-*VENUS*. The p*EIN2* and p*EIN3* fragments (2121 bp and 1900 bp respectively) for the p*EIN2*:*VENUS* and p*EIN3*:*VENUS* transgenes were cloned from the genomic DNA of wild type (Col-0). The involved primer sequences are listed in Supplementary Table S1. The purified DNA fragments were ligated and inserted into the modified vector, pCAMBIA1300-*VENUS*, using the ClonExpress II One Step Cloning Kit (Vazyme, Nanjing, China). The p*DYT1* used in this study was the p*DYT1*-550 reported previously [27]. The *EIN2,* and *EIN3* clones for the p*DYT1*:*EIN2* and p*DYT1*:*EIN3* constructs were amplified from the genomic DNA of Col-0. Relevant primer sequences are listed in Supplementary Table S1. All constructions were sequenced, transformed into *Agrobacterium tumefaciens* strain GV3101 and then transformed into *Arabidopsis* using the floral dip method.

### 2.5. Real-Time PCR Analyses

Total RNA was extracted from inflorescences of single T1 transgenic plants or *can1* and *can2* mutants using the TRIzol kit (Invitrogen, Carlsbad, CA, USA) following the manufacturer’s instructions. The total RNA was then reverse transcribed into cDNA according to the manufacturer’s instructions (TransGen Biotech, Beijing, China). Quantitative real-time PCR was performed using the ABI 7300 System with the SYBR Green Real-Time PCR Master Mix (Toyobo, Osaka, Japan). All genes were normalized to β-tubulin, and three biological replicates were performed per experiment. Relevant primer sequences are listed in Supplementary Table S1.

## 3. Results

### 3.1. Exogenous Ethylene Treatment Causes Filament Shortening, Pollen Abortion and Leads to Male Sterility

**Figure 1:**
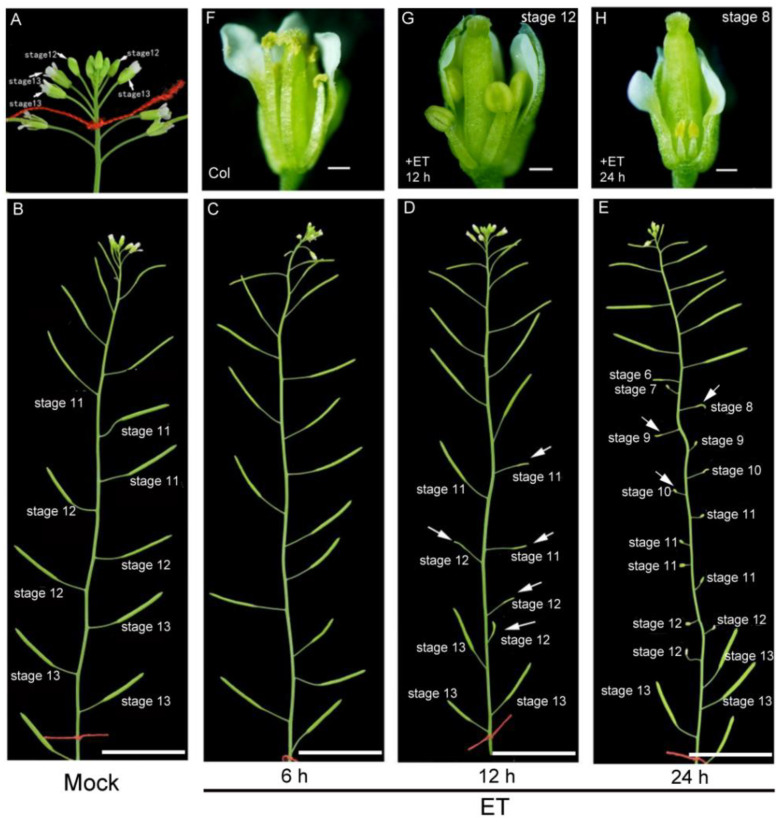
The fertility was disturbed after exogenous ethylene treatment. (**A**) Inflorescence before ethylene treatment. Note that the start position of inflorescence (flower with anthers at stage 13) was marked with a red line so as to observe the elongation of siliques after 14 days. (**B**–**E**) Inflorescence of Col without ET treatment (**B**), with ethylene (100 µL L^−1^) treatment for 6 h (**C**), 12 h (**D**), and 24 h (**E**). After ET treatment, these plants grew for 14 days under normal conditions. Arrows indicate the closed buds or short siliques without seeds. The developmental stage of anthers in each bud before ethylene treatment is marked next to the corresponding siliques or flower buds. Bars = 2 cm in B–E. (**F**) Col flower. Bar = 1.0 mm. (**G**,**H**) Col flower after ethylene (100 µL L^−1^) treatment for 12 h (**G**) and 24 h (**H**). Note that filaments are shortened after 12 h or 24 h ethylene treatment, and the development of the anther of stage 8 is abnormal after 24 h ethylene treatment. Bars = 1.0 mm in (**G**,**H**).

To investigate the detailed effect of ethylene on the stamen, we carried out an ethylene treatment experiment in *Arabidopsis*. The process of anther development has been divided into 14 stages according to structure properties [21]. Based on each flower position and the DAPI staining results of its microspores from more than three inflorescences, we can judge the approximate developmental stage of the anther by its position in an inflorescence (Figure 1A). We treated the wild type (Col-0) with ethylene (100 µL L^−1^) for 6 h, 12 h, or 24 h, respectively, then observed the fertility of each plant after 14 days of growth. Compared with the Col plants after mock treatment (Figure 1B), plants treated with ethylene for 6 h had basically normal fertility (Figure 1C). Plants treated with ethylene for 12 h or 24 h showed that some siliques failed to produce seeds (Figure 1D,E and Figure S1). Artificially pollinated viable pollens from untreated plants to the ethylene-treated stigma could produce normally elongated siliques, indicating that the sterility phenotype did not result from female defects. The anthers in the flower bud that correspond to these short siliques were from stage 11 to stage 12 (for 12 h treatment) and from stage 6 to stage 12 (for 24 h treatment) (Figure 1D,E). Dissection of these flower buds after ethylene treatment revealed shortened filaments (Figure 1G,H) and abnormal anthers (Figure 1H).

**Figure 2:**
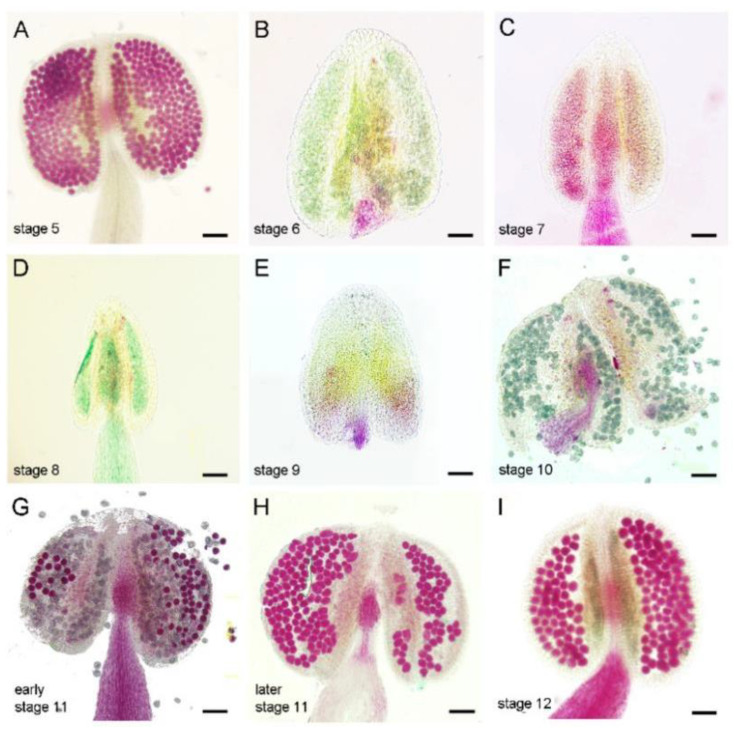
Alexander staining of anthers after 24 h of ethylene treatment. (**A**–**I**) Alexander staining of stage 5 to stage 12 anthers after 24 h treatment with ethylene (100 µL L^−1^). Bars = 50 µm. After ethylene treatment, plants were transferred to normal condition for further growth. The flower buds in the inflorescence gradually mature, and Alexander staining was carried out to stain the anthers of each mature flower.

We then carried out Alexander staining to detect pollen activity for anthers at different stages after ethylene treatment. After ethylene treatment, the pollen activity was not disturbed in the anthers at stage 5 or younger than stage 5, which was consistent with the normal fertility of these buds (Figure 1 and Figure 2A). While in ethylene-treated anthers from stage 6 to stage 10, no viable pollen was observed in the anther when it should be mature (Figure 2B–F), indicating that the anthers at this period are very sensitive to ethylene treatment. For anthers at early stage 11, ethylene induces some pollen abortion (Figure 2G). At later stage 11 and stage 12, anthers could produce normal pollens (Figure 2H,I and Figure S1), indicating that these flowers’ sterility phenotype resulted from the filament’s elongation inhibition. We thus conclude that ethylene treatment inhibits filaments elongation and leads to pollen abortion. The sensitivity of anthers that respond to ethylene is dependent on specific stages. For the anthers younger than stage 5 or older than stage 11, ethylene treatment could not disturb the following pollen development. Anthers from stage 6 to early-stage 11 were mostly sensitive to ethylene treatment, showing complete or partial loss of pollen activity. Because the anther defects induced by the 24 h treatment of ethylene are similar to the phenotype of the female flowers in cucumber, we conducted the following ethylene treatment experiments with this condition.

### 3.2. Ethylene Treatment Leads to Degeneration of the Tapetum and Microspores

**Figure 3:**
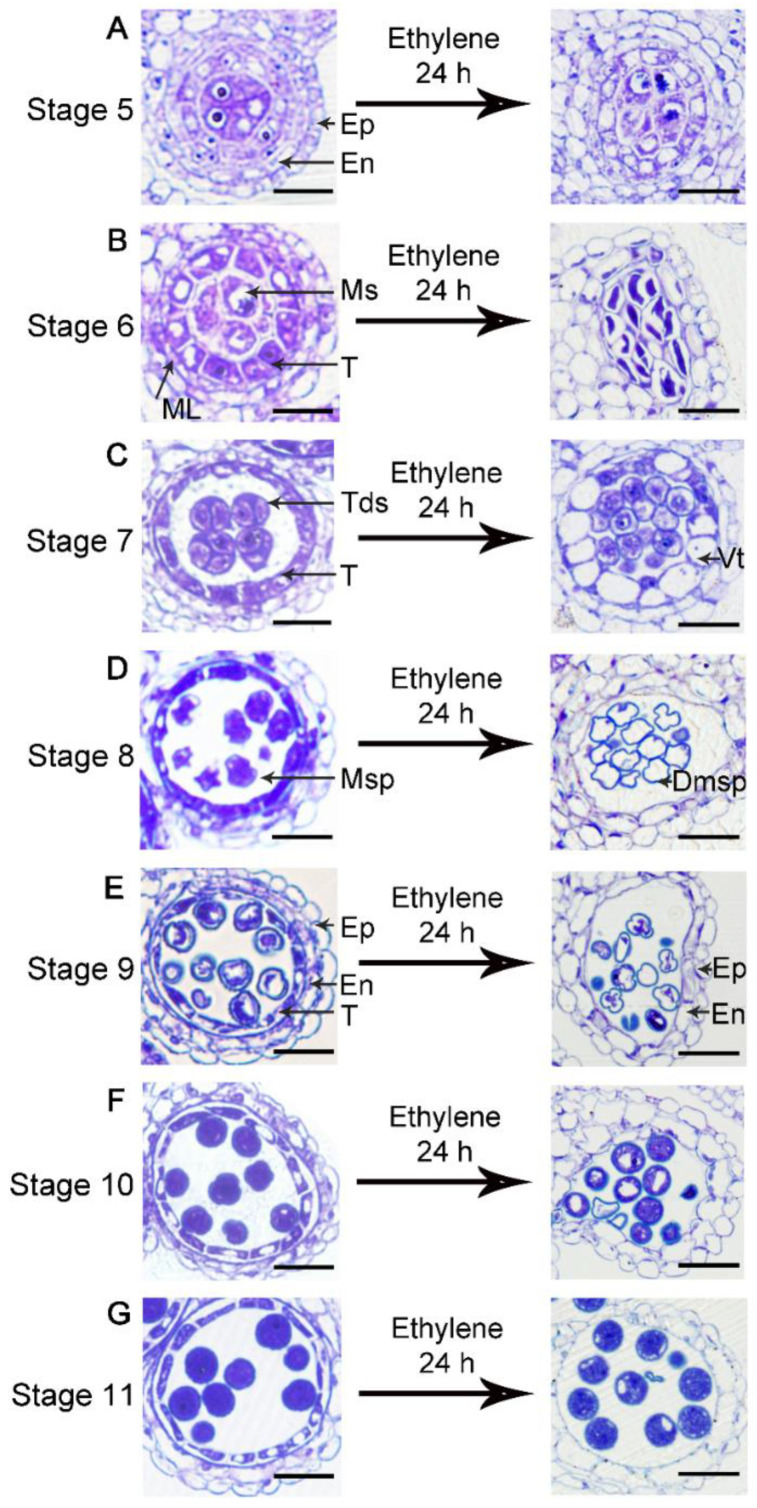
Ethylene treatment resulted in the degeneration of tapetum and microspores. (**A**–**G**) Semi-thin sections of Col anther before and after ethylene treatment (100 µL L^−1^ for 24 h). Note for the degeneration of tapetal layer and microspores. Bars = 20 µm. Ms: microsporocyte; Tds: tetrad; Msp: microspores; Ep: epidermis; En: endothecium; ML: middle layer; T: tapetum; Vt: Vacuolated tapetum; Dmsp: degenerated microspores.

To further determine the effects of ethylene on each cell layer of the anther, we made semi-thin sections to observe the cytological phenotypes of anthers at different stages after ethylene treatment. According to the time course of anther and pollen development (Figure S2) [28], after 24 h, anthers may develop into the next developmental stage. At stage 5, the four cell layers and the microsporocytes are differentiated and can be identified (Figure 3A). After ethylene treatment for 24 h, all the cell layers at this stage were normal (Figure 3A), and the microsporocytes seemed to normally enter meiosis as that of stage 6 (Figure 3B). The phenotype at this stage is consistent with the normal pollen activity observed after ethylene treatment (Figure 2). For the anther at stage 6, the tapetum disappeared after treatment, and partially degenerated microsporocytes or tetrads were observed in the locule (Figure 3B). After meiosis, tetrads composed of four haploid microspores are normally formed in the anther locule at stage 7 (Figure 3C). After treatment, the tapetal cells became severely vacuolated, and the microspores were released from the tetrad (Figure 3C). The tapetum undergoes programmed cell death (PCD) in the following stages to provide the material for pollen development and pollen wall formation, and the microspore undergoes two rounds of mitosis (Figure 3D–G). In contrast, ethylene-treated anthers from stage 8 to stage 10 also showed premature degeneration of the tapetal layer, and the microspores were gradually degraded (Figure 3D–F). At stage 11, the tapetum degenerate into a very thin layer in Col (Figure 3G). After ethylene treatment, the tapetum disappeared at stage 11, while most of the microspores at this stage appeared normal (Figure 3G), which was consistent with the Alexander staining results. From these results, we concluded that ethylene treatment induced the severe degeneration of the tapetum layer and microsporocytes or microspores from stage 6 to stage 10.

**Figure 4:**
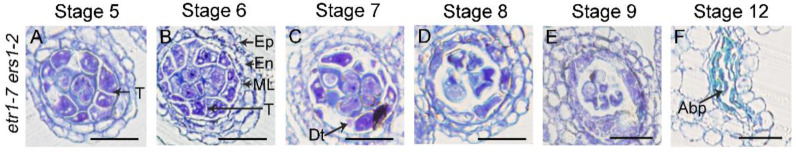
*etr1-7 ers1-2* double mutant showed abnormal tapetum and microspores. (**A**–**F**) Semi-thin sections of anthers from stage 5 to stage 12 of *etr1-7 ers1-2* mutant. Bars = 20 µm. Ep: epidermis; En: endothecium; ML: middle layer; T: Tapetum; Dt: Degenerated tapetum; Abp: Aborted pollen.

In the ethylene signaling pathway, ETR1 and ERS1 are two major receptors. We also made semi-thin sections of the ethylene receptor double mutant *etr1-7 ers1-2*, which exhibited some constitutive ethylene responsiveness and leads to male sterility [19]. Compared with normal anther development (Figure 3A–G), *etr1-7 ers1-2* showed overall expansion of the tapetal layer at stage 5 and stage 6 (Figure 4A,B). At stage 7, the regular shape of the tetrad was not observed in the locule and the tapetum became fragmentary, implying the abnormal degradation of the tapetal cells (Figure 4C). In the following stages, the microspores gradually degenerated into irregular shapes (Figure 4D–F). Consequently, the defects in the anther of *etr1-7 ers1-2* are similar to that of ethylene-treated wild type, although the phenotypes are much weaker. These results indicate that the ethylene content and its signaling pathway should be strictly controlled for normal anther development.

### 3.3. The Anther Defects Induced by Ethylene Treatment Are EIN2- and EIN3/EIL1-Dependent

**Figure 5:**
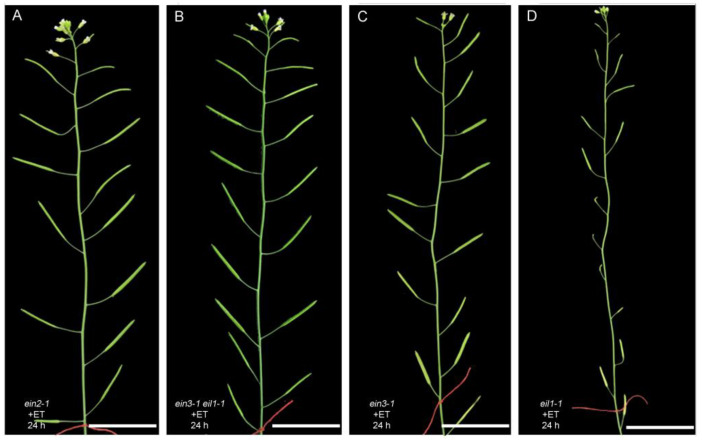
The inflorescence of *ein2-1* and *ein3-1 eil1-1* is insensitive to exogenous ethylene treatment. (**A**–**D**) The inflorescence of *ein2-1* (**A**), *ein3-1eil1-1* (**B**), *ein3-1* (**C**) and *eil1-1* (**D**) after treated with ethylene (100 µL L^−1^) for 24 h. After ethylene treatment, these plants were transferred to normal growth condition for 14 days. Bars = 2 cm.

To further identify components of the ethylene signaling pathway that mediate the anther’s repression, we treated *ein2-1*, *ein3-1*, *eil1-1* single mutants and *ein3-1 eil1-1* double mutants with ethylene (100 µL L^−1^) for 24 h and observed the fertility of these plants. We found that *ein2-1* and *ein3-1 eil1-1* exhibited ethylene-insensitive phenotypes with commonly elongated siliques (Figure 5A,B), whereas *ein3-1* and *eil1-1* single mutant plants showed different degrees of ethylene-sensitive phenotypes with fewer seeds or no seeds in siliques (Figure 5C,D). *ein3-1* showed less sensitivity to ethylene than *eil1-1*, indicating that *EIN3* plays a more important role than EIL1 in anther repression. We thus determined that the anther defects induced by ethylene are mediated by *EIN2* and *EIN3*/EIL1.

### 3.4. Exogenous Ethylene Treatment Activates EIN2 and EIN3 Transcription in Tapetum

**Figure 6:**
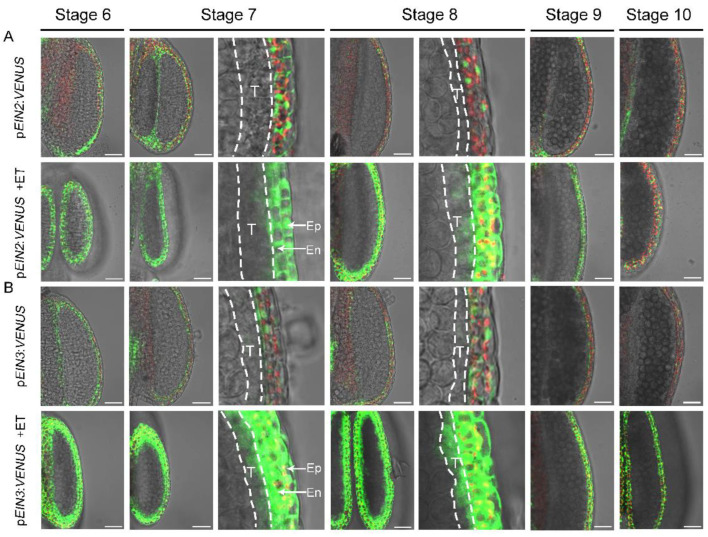
The expression patterns of p*EIN2*:*VENUS* and p*EIN3*:*VENUS* reporters during anther development. (**A**,**B**) Fluorescence images of the p*EIN2*:*VENUS* (**A**) and p*EIN3*:*VENUS* (**B**) before and after ethylene treatment. The dashed box in the close-up region indicate the tapetum layer in both (**A**,**B**). Note for the VENUS signals were appeared in tapetum and were enhanced in epidermis and endothecium after ethylene treatment. The green channel showed the VENUS signals and the red fluorescence channel showed auto-fluorescence of chlorophyll in epidermis (Ep) and endothecium (En). T: tapetum. Bars = 30 μm.

The temporal and spatial expression of *EIN2* and *EIN3* during anther development was investigated involving the p*EIN2*:*VENUS* and p*EIN3*:*VENUS* transgenic plants. The VENUS signal for p*EIN2*:*VENUS* and p*EIN3*:*VENUS* was observed in both the anther wall and filaments (Figure 6A,B and Figure S3). The expression peak of *VENUS* was observed at stage 6 and stage 7, and the fluorescence intensity is gradually decreased in the following stages (Figure 6A,B). Both *EIN2* and *EIN3* showed similar expression patterns during anther development. Such transcriptional patterns are correlated well with their function in anther repression and filament inhibition. We noticed that the VENUS signal in both p*EIN2*:*VENUS* and p*EIN3*:*VENUS* accumulated in the outer layers of the anther wall such as the epidermis and endothecium, but not in the tapetum and microspores (Figure 6A,B). As results had shown that the tapetal cells severely degenerated after ethylene treatment (Figure 3),this prompted us to investigate whether the *EIN2* and *EIN3* expression are activated in the tapetum by ethylene treatment. Therefore, we observed the VENUS fluorescence of the transgenic plants after ethylene treatment for 24 h. In both kinds of transgenic plants, the VENUS signal intensity was significantly increased in the anther wall after treatment (Figure 6A,B). More importantly, the VENUS fluorescence was detected in the tapetum (Figure 6A,B) but not in microspores. These results indicated that the induction of *EIN2* and *EIN3* are activated in the tapetal cell after ethylene treatment.

**Figure 7:**
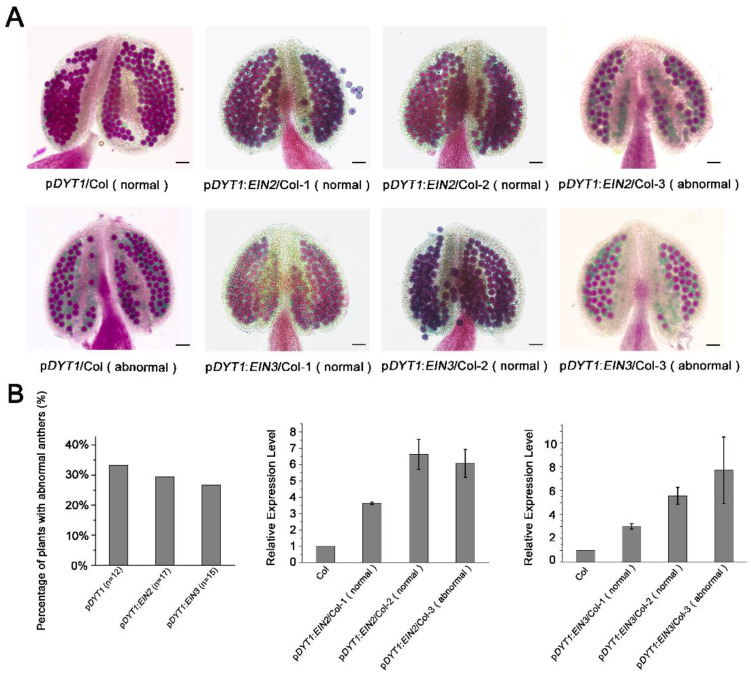
Ectopic transcription of *EIN2* and *EIN3* in tapetum did not influence pollen formation. (**A**) Alexander staining of pollens of p*DYT1*/Col, p*DYT1*:*EIN2*/Col, and p*DYT1*:*EIN3*/Col transgenic plants. Note, all of the three transgenic populations showed two phenotypes: plants showed normal pollens and plants showed partial pollen lethality phenotype. Bars = 50 µm. (**B**) Left panel: The percentage of plants with abnormal anthers in each group of the transgenic population. Middle panel: Real-time quantitative RT-PCR analysis of *EIN2* transcript levels in three independent lines of p*DYT1*:*EIN2*/Col transgenic plants. Right panel: Real-time quantitative RT-PCR analysis of *EIN3* transcript levels in three independent lines of p*DYT1*:*EIN3*/Col transgenic plants. β-tubulin was used as an internal control for normalization. Data are mean ± SEM of three biological replicates with technical duplicates for each.

Because ethylene can activate the transcriptions of *EIN2* and *EIN3* in the tapetum and induce tapetum degradation, we wondered whether artificially overexpressing these two genes in the tapetum can cause anther defects similar to ethylene treatment. We used a tapetum-specific promoter, *DYT1,* to ectopically express *EIN2* and *EIN3* in the tapetal layer around stage 6 [27]. We obtained 17 and 15 independent T1 lines of p*DYT1:EIN2*/Col and p*DYT1:EIN3*/Col transgenic plants, respectively, and most of these lines (12/17 of p*DYT1:EIN2*/Col and 11/15 of p*DYT1:EIN3*/Col) showed normal fertility with anthers full of pollen grains (Figure 7). The few remaining plants showed a partial pollen lethality phenotype (Figure 7A). We found that the percentage of plants showing the partial pollen defects in the p*DYT1:EIN3*/Col and p*DYT1:EIN2*/Col transgenic population was not significantly different from that of the transgenic control p*DYT1*/Col (4/11) (χ^2^ < χ^2^_0.05,1_ = 3.84 *p* > 0.05) (Figure 7B), implying that the pollen defect phenotype is independent of the construct used for transformation, which is a common feature occurring in transgenic populations reported previously [29]. We next detected the transcripts level of *EIN2* or *EIN3* in the transgenic plants and found three to six-fold increased transcripts of *EIN2* or *EIN3* in each T1 line (Figure 7B), confirming the activity of the *DYT1* promoter. These results showed that only increasing the transcriptions of *EIN2* and *EIN3* did not influence the anther development, indicating that the expression pattern and function of these two factors are also regulated from other levels.

### 3.5. Exogenous Ethylene Treatment Can Stabilize the Accumulation of EIN3 in Tapetum

**Figure 8:**
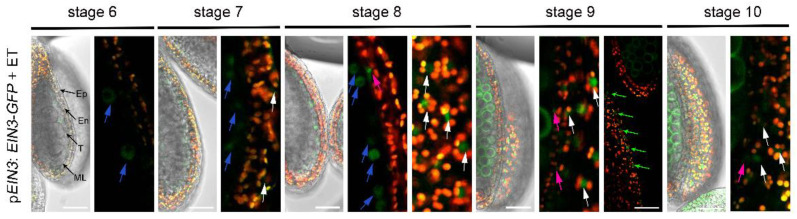
The expression pattern of *EIN3* in anthers after ethylene treatment. Fluorescence images of the p*EIN3*:*EIN3-GFP* in anthers. The green channel showed the GFP signal and the red fluorescence channel showed auto-fluorescence of chlorophyll in epidermal and endothecium. The blue, white, and red arrows indicate the GFP signal in the nuclei of tapetum, epidermis and endothecium, respectively. The green arrow indicate the GFP signal in the nuclei of filament. Ep: epidermis; En: endothecium; ML: middle layer; T: tapetum. Bars = 30 μm.

It has been reported that *EIN3* is regulated at the post-translational level. In the absence of ethylene, *EIN3* is degraded by two F-box proteins, EBF1 and EBF2 [30,31,32]. To investigate the expression pattern of *EIN3* during anther development, we observed the GFP signal of the p*EIN3:EIN3-GFP*/Col transgenic plants before and after ethylene treatment. Under normal growth conditions, no significant GFP signal could be detected in the anther from stage 5 to stage 12 of p*EIN3:EIN3-GFP*/Col transgenic plants (Figure S4). After ethylene treatment, *EIN3* proteins were detected in the anther (Figure 8). At stage 6, a weak GFP signal was detected in a few cells of tapetum. At stage 7 and 8, a strong GFP signal was detected in most of the tapetal cells. In addition to its accumulation in the nuclei of tapetal cells, *EIN3*-GFP was also observed in the nuclei of the epidermis and endothecium, and in the nuclei of filaments (Figure 8). These results indicated that ethylene not only activates the transcription of *EIN3*, but also stabilized *EIN3* in the tapetum.

### 3.6. The Expression of Several ERFs and SAGs Genes Are Induced by Ethylene and the Expression of the Five Key Transcription Factor Genes in Tapetum Development Is Reduced after Ethylene Treatment

It has been proposed that an ethylene inducible calcium dependent nuclease Cs*CaN* may be required for the DNA damage of anther primodia in the female flower in cucumber [20]. There are two homologs of Cs*CaN* in *Arabidopsis*, *CaN1* and *CaN2*. To verify whether the *CaN* genes are also involved in anther repression in *Arabidopsis* after ethylene treatment, we first obtained mutants with T-DNA insertion at *CaN1* or *CaN2* locus, named *can1-1* and *can2-1*, in which the expression level of the corresponding genes decreased sharply (Figure S5). We then generated *can1-1 can2-1* double mutant by crossing the two single mutants. *can1-1*, *can2-1* and *can1-1 can2-1* mutants do not cause a noticeable defect during growth. We applied ethylene (100 µL L^−1^) to treat the *can1-1*, *can2-1* single mutants and *can1-1can2-1* double mutants for 24 h. We found that they all showed the ethylene responsive phenotype reminiscent of the ethylene-treated wild type (Col-0) (Figure S5). Therefore, we proposed that the function of *CaNs* may not be conserved in ethylene-induced anther repression in *Arabidopsis* and cucumber. Alternatively, other genes that play redundant roles with *CaNs* may also be required during this process in *Arabidopsis*.

**Figure 9:**
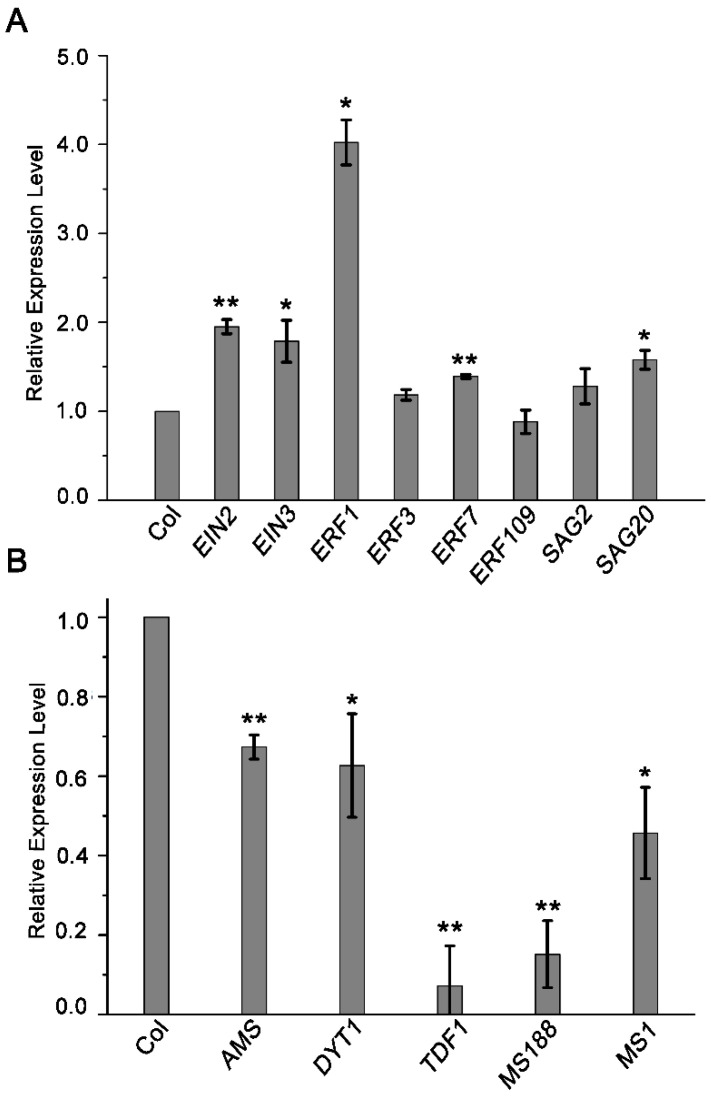
Quantitative RT-PCR analysis of *ERFs*, *SAGs* and tapetum development-related key transcription factors after ethylene treatment. (**A**) Relative expression of *EIN2*, *EIN3*, *ERFs* and *SAGs* genes in the inflorescence of Col after ethylene treatment for 24 h. (**B**) The expression of the five key transcription factor genes in tapetum development is reduced after ethylene treatment. Relative expression of *AMS*, *DYT1*, *TDF1*, *MS188*, and *MS1* in the inflorescence of Col-0 after ethylene treatment for 24 h. Col treated with air for 24 h was used as a mock. Gene expression was relative to that of the mock. β-tubulin was used as an internal control for normalization. Data are mean ± SEM of 3 biological replicates with technical duplicates for each. * *p* < 0.05, ** *p* < 0.01 (*t*-test).

Ethylene-responsive element binding factors (ERF) family of transcription factors were originally identified in tobacco by their ability to recognize a conserved ethylene response element (GCC) box [33]. ERF1 can regulate the expression of a large number of genes responsive to both ethylene and jasmonate [34]. The expression of several *ERF*s has been proven to be increased after ethylene treatment [34,35]. *SENESCENCE-ASSOCIATED GENE*s (*SAG*s) are up-regulated in senescing leaves and expression of several *SAG*s can be induced by exogenous ethylene in leaves [36,37]. The expression of *SAG20* is increased during the flower senescence in *Camellia lutchuensis* [38]. The transcripts of several *ERF*s and *SAG*s genes such as *ERF3*, *ERF7*, *ERF109*, *SAG2*, and *SAG20*, have been detected in the tapetal cells (Table S2) [39]. We analyzed the transcript level of these five genes, *ERF1*, *EIN2*, and *EIN3* before and after ethylene treatment to confirm whether the expression of these genes respond to ethylene treatment in inflorescence. Consistent with the increased expression of *VENUS* in p*EIN2:VENUS* and p*EIN3:VENUS* plants after ethylene treatment (Figure 6), the transcription of *EIN2* and *EIN3* was increased with ethylene treatment (Figure 9A). As shown in Figure 9, expression of *ERF1*, and to a lesser extent of, *ERF7* and *SAG20*, was significantly increased, whereas the expression of *ERF3*, *ERF109* and *SAG2* was unchanged after ethylene treatment. In *Arabidopsis*, five tapetum specifically expressed transcription factors, DYSFUNCTIONAL TAPETUM 1 (DYT1), DEFECTIVE IN TAPETAL DEVELOPMENT AND FUNCTION 1 (TDF1), ABORTED MICROSPORE (AMS), MS188/MYB103/MYB80, and MALE STERILITY 1 (MS1), are essential for tapetum development and pollen formation [40]. Mutations in each of these five genes lead to tapetum defects and pollen abortion. Consistent with the observed tapetum defects, the expression of these five genes were decreased to a different extent after ethylene treatment (Figure 9).

## 4. Discussion

Under the ethylene treatment condition used in this study, stamens but not pistils displayed obvious developmental defects. This is similar to the performance of cucumber to produce female flowers in the agricultural application of ethylene. We can know from previous reports that female fertility defects also appeared in the *etr1 ers1* double mutants in *Arabidopsis* [19]. Ethylene is a gaseous hormone and can diffuse from cells to cells. We speculate that it may be much easier for ethylene to diffuse into anthers than ovules as they are contained within two fused carpels in *Arabidopsis*. Compared with pistils, stamens are much more sensitive to ethylene. Regardless of the short-termed or long-termed ethylene treatment, filaments became significantly shortened (Figure 1). *EIN2* and *EIN3* expression was consistently significantly higher in filaments (Figure S3). The classic ethylene triple-response phenotype for etiolated seedling includes the inhibition of root and hypocotyl elongation, swelling of the hypocotyl and exaggeration of the curvature of apical hooks in *Arabidopsis* [41]. Filament shortening is similar to that of hypocotyl, although the underlying mechanism remains to be explored.

By detailed phenotype analysis, we also observed that anthers at different developmental stages have different responses to ethylene. For the anthers from stage 6 to 10, the tapetum and microspores are almost degenerated (Figure 3). During this period, the development of pollen depends on various nutrient, pollen wall materials and kinds of enzymes provided from the tapetum [42,43,44,45]. Premature or delayed PCD will lead to pollen defects [46,47]. In addition to exogenous ethylene application, defects in tapetal and pollen development are also observed in the *etr1 ers1* mutants (Figure 4), indicating that the ethylene content and its signaling pathway need to be precisely regulated to ensure the normal development of tapetum and pollen. Interestingly, the pollen activity of the anthers older than stage 11 was not disturbed after ethylene treatment. Several critical biological processes have been finished or almost completed at these stages. For example, the nuclear divisions of pollens are finished at stage 11, so the pollens enter the three-celled stage after this stage [21]. At the meantime, the exine and intine layers of the pollen wall have been established, and the pollen coat has appeared in the sculpture cavities of exine from stage 11 [42,48]. In addition, the tapetum has largely degenerated at stage 11 under normal growth conditions [21]. It is reasonable to believe that the pollen activity at these stages may not strongly be dependent on the function of the surrounded sporophytic cell layers such as tapetum. Therefore, the degeneration of tapetal cells cannot significantly affect the maturation and activity of pollens after stage 11. In conclusion, the sensitivity of anther response to exogenous ethylene is dependent on specific developmental stages. In the female flower of cucumber, the anther repression is initiated from the anther primordium stage [9,10]. This stage is much earlier than the anther development period (stage6-stage10) sensitive to ethylene in *Arabidopsis*, indicating that the time point of ethylene inhibiting the stamen development is different in cucumber and in *Arabidopsis.*

Our experimental results proved that the stamen developmental defects caused by exogenous ethylene are dependent on the function of *EIN2* and *EIN3*/EIL1. Consistently, the expression of *EIN2* and *EIN3/EIL1* was detected in the stamen (Figure 6 and Figure 8). After ethylene treatment, the expression of *EIN2* and *EIN3* was detected in the tapetum, but not in microsporocytes or microspores, indicating that ethylene inhibits anther development mainly by activating the *EIN2*-*EIN3*/EIL1 signaling pathway in the tapetum. The tapetum layer is particularly sensitive to ethylene treatment. After ethylene treatment, filaments and other cell layers in the anther wall such as the epidermis and endothecium can still maintain the basic cell structure, while the tapetum is almost degraded. Under normal growth conditions, the tapetal cell undergoes degradation by PCD. Although the detailed mechanism is unclear, we speculate that the hypersensitivity of the tapetum to ethylene may be related to the destined PCD event. Our results demonstrated that ethylene can regulate the expression of *EIN3* in the tapetum from two aspects: transcriptional activation and protein stability maintenance (Figure 6 and Figure 8). The activated *EIN2*-*EIN3*/EIL1 signaling pathway may then lead to premature PCD and induces the degeneration of the tapetum. Here, we showed that the expression of the five key transcription factors that play essential roles for tapetum development was reduced after ethylene treatment (Figure 9). We also demonstrated that the expression of *ERF1*, *ERF7*, and *SAG20* was induced in the inflorescence after ethylene treatment (Figure 9). The changed expression of these genes is consistent with the anther defects induced by ethylene.

It has been reported that *EIN2*, *EIN3*, and EIL1 are required for the normal degeneration of synergid cells in the embryo sac during fertilization [6,7,8]. Since the tapetal cell undergoes PCD under normal growth conditions, and exogenous ethylene can induce the premature of PCD, we wondered whether the ethylene signaling pathway is also required for the normal PCD of tapetum. In the *ein3-1* and *eil1-1*, pollens can be normally formed (Figure S6), implying that the function as well as the PCD program of tapetum are not disturbed in the mutants, indicating that *EIN3* and EIL1 may not be essential for the PCD of tapetum under normal conditions. Consistently, neither the transcripts nor the protein of *EIN3* could be detected in this cell layer under normal growth conditions (Figure 6 and Figure 8). What then is the function of preparing transcripts of the key factors of the ethylene signaling pathway such as *EIN2*, and *EIN3* in the outer layers (epidermis and endothecium) of the anther wall (Figure 6)? We speculate that such preparation may be required for the anther to respond to kinds of stresses quickly. Previous articles supported that environmental stresses such as heat, shade, flooding, and water deficiency can trigger ethylene production [49,50,51]. In the meantime, *SAG20* was reported to respond to ozone treatment or the treatment of Nep1, a fungal protein that causes necrosis [52,53]. When plants encounter stress or exogenous ethylene treatment as in this paper, ethylene may activate the *EIN2*-*EIN3*/EIL1 signaling pathway to quickly disturb or block stamen development, and may transfer more energy from the reproductive process to stress resistance.

In conclusion, (1) we found that the sensitivity of anther and pollen response to exogenous ethylene is dependent on specific developmental stages. Ethylene treatment leads to shortened filaments and severe degeneration of the middle layer, tapetum and microspores from anther stage 6 to stage 10. (2) We provide evidence that all the defects induced by ethylene are dependent on the canonical ethylene signal pathway mediated by *EIN2* and *EIN3*/EIL1 in vivo. Consistently, ethylene not only activates the transcription of *EIN2* and *EIN3*, but also induces the stabilization of *EIN3* in the tapetum. (3) This work clarifies that the ethylene signaling pathway is not required for normal PCD of tapetum but it should be strictly blocked during anther development, especially in the tapetum. Whether this signaling pathway in anther can respond to various stresses and is necessary for stress resistance requires further study.

## Data Availability

Not applicable.

## Supplementary Materials

The following supporting information can be downloaded at: https://www.mdpi.com/article/10.3390/cells11193177/s1, Figure S1: The closed flower and Alexander staining of anthers after ethylene treatment; Figure S2: The time course of anther development; Figure S3: *EIN2* and *EIN3* are transcribed in filaments; Figure S4: Expression pattern of *EIN3* in anthers; Figure S5: The fertility of the *can* mutants is disturbed after exogenously ethylene treatment; Figure S6: Alexander staining of pollens of *eil1-1* and *ein3-1*; Table S1: Primers used in the study; Table S2: The transcripts of *ERF*s and *SAG*s were detected in the tapetum.
