# Supplementary material for: Ethylene Activates the EIN2-EIN3/EIL1 Signaling Pathway in Tapetum and Disturbs Anther Development in Arabidopsis
Source: Cells. 2022 Oct 10;11(19):3177. doi: 10.3390/cells11193177 (PMC9563277; doi:10.3390/cells11193177)
Supplement: Supplementary file 1 [file cells-11-03177-s001.zip › cells-1857213-supplementary.pdf]

Supplementary materials

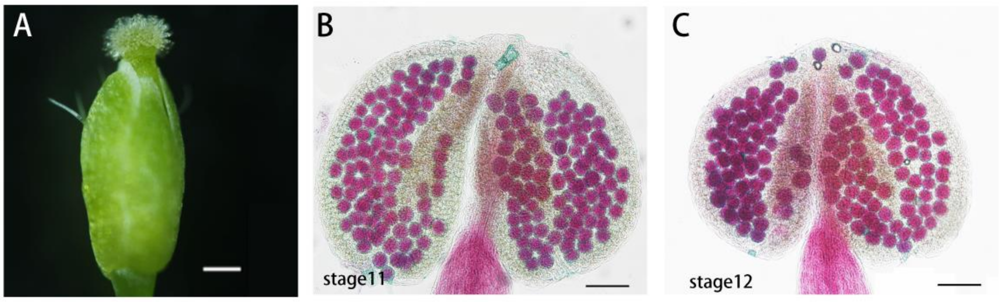

Figure S1. The closed flower and Alexander staining of anthers after ethylene treatment. (A) Flower was closed after ethylene treatment for 24 h. Bar = 1.0 mm. (B-C) After 12 h of ethylene treatment, Alexander staining of anther at stage 11 (B) and stage 12 (C). Note that the anthers are full of viable pollens. Bar = 50 μm in B and C.

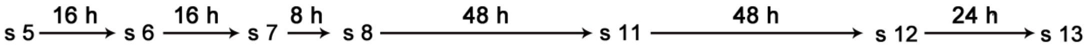

Figure S2. The time course of anther development. These data were extracted from a published paper [28].

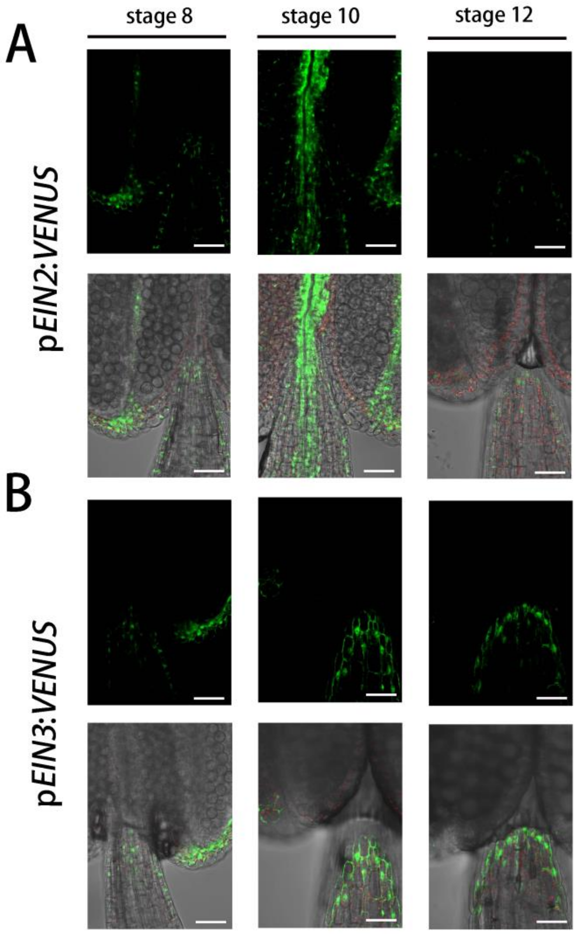

Figure S3. ***EIN2*** **and** ***EIN3*** **are transcribed in filaments.** (A-B) The filaments expression of p*EIN2:VENUS* (A) and p*EIN3:VENUS* (B) reporters during stamen development. Bars = 30 µm.

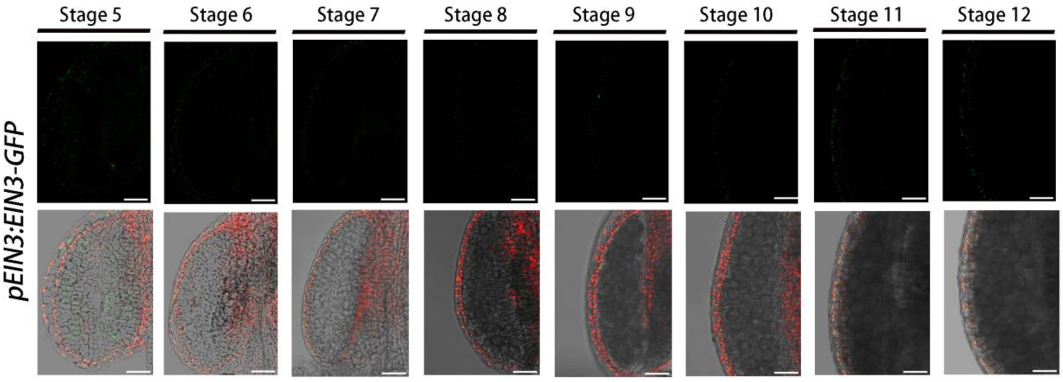

Figure S4. Expression pattern of EIN3 in anthers. The green channel showed the GFP signal and the red fluorescence channel showed auto-fluorescence of chlorophyll. Bars=30µm.

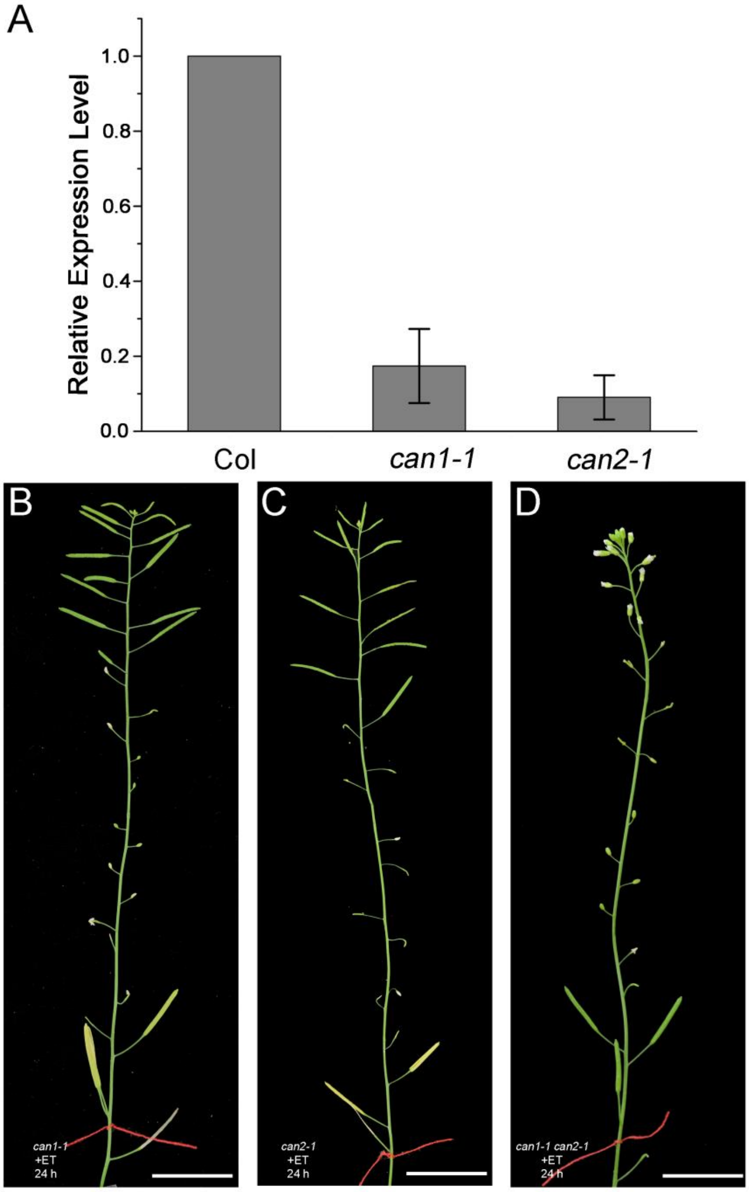

Figure S5. The fertility of the *can* mutants is disturbed after exogenously ethylene treatment. (A) Real-time quantitative RT-PCR analysis of the transcript levels of *CaN1* and *CaN2* in *can1-1* and *can2-1*, respectively. (B-D) The inflorescence of *can1-1* (B), *can2-1* (C) and *can1-1can2-1* (D) after treated with ethylene (100 μL L^{-1}) for 24h. After ethylene treatment, these plants grew for 10-14 days under normal condition. Note for the closed buds or short siliques without seeds, indicating these *can* mutants all showed ethylene-sensitive phenotype.

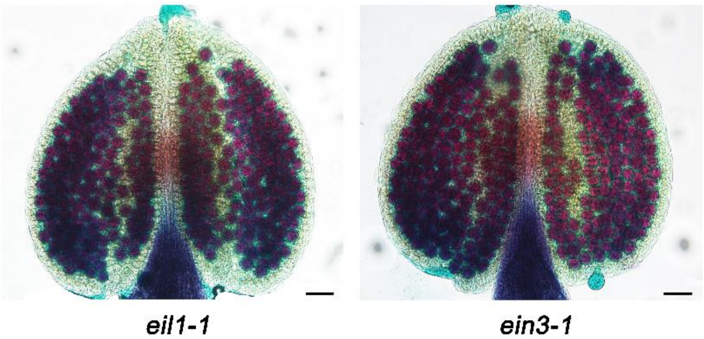

Figure S6. Alexander staining of pollens of *eil1-1* and *ein3-1*. Note that the anthers are full of viable pollens. Bars = 50 μm.

Table S1. Primers used in the study.

Primer name	Sequence（5'-3'）	Function
ein2-1	CCAGAGGAAAGAGAGTTGGATGTAAAGTACTCTACCGCT	
ein2-1	CGCCATCTTTGTTTCAACAATCAGATCC	
ein3-1-F	TACCAAGTATCAAGCGGAG	
ein3-1-R	AGGCCACCAATCCTCTTTC	
eil1-1-F	GGGAATGGTGGAAAGATAAG	
eil1-1-R	CTTTCGCCGTCATCTTATCC	
can2-1-LP	GGTTGATGTGATCGAAAGAATC	Mutant identification
can2-1-RP	GGGCTCTTAAAGATTGTTGGG	
LBa1	TGGTTCACGTAGTGGGCCATCG	
ers1-2-F	GTGCCGTCTCGGGATAACAAACTTTCTAT	
ers1-2-R	TCGAGCATGTACTGCCATCTCAGCCTCTT	
etr1-7-F	GCAATTGTATTGAACCGCA	
etr1-7-R	CAACCTAGCCACAGGAGAA	
can1-1-LP	TCCCACTGAATTCAAAACACC	
can1-1-RP	GAAGCCTCCAGGCTTGTTATC	
LB1.3	ATTTTGCCGATTTCGGAAC	
p*EIN2*-Venus-F	gaattcgagctcggtacccggggatccAGTTTTTGATTTCTCTTTTATT	*EIN2* promoter
p*EIN2*-Venus -R	gcccttgctcaccattctagaCCTAAATCTATCTGATAATATAATT	
p*EIN3*-Venus -F	gaattcgagctcggtacccggggatccCAACCCTGAAAAAAAAGATAACC	
p*EIN3*-Venus -R	gcccttgctcaccattctagaTGTAACCTGTAACAAATCAAATACAC	*EIN3* promoter
p*DYT1*-F	gagctcGACCTTGAAGAAGAAGTGACCG	
p*DYT1*-R	ggtaccTTATTTCTTCTTCTTTGATAATTTTTCAGA	*DYT1* promoter
p*DYT1*-*EIN2*-F	ttatcaaagaagaagaaataacggATCTCTCTCTTTCGATGGAACTGA	
p*DYT1*-*EIN2*-R	acgctgcaggtcgactctagaggatccCTAGCGTCGATGTGTCAGCTAC	*EIN2* gene
p*DYT1*-*EIN3*-F	ttatcaaagaagaagaaataacggAGTATATATACTATGATCTATCTTCC	
p*DYT1*-*EIN3*-R	acgctgcaggtcgactctagaggatccAAGAGAGCTTGCAAATATAT	*EIN3* gene
qRT-*TUB*-F	GATTTCAAAGATTAGGGAAGAGTA	
qRT-*TUB*-R	GTTCTGAAGCAAATGTCATAGAG	
qRT-*CAN1*-F	CAGAATGAGGAAGTGCTTGCT	
qRT-*CAN1*-R	CTACACATCTTCCATAACGATCC	
qRT-*CAN2*-F	GCTTGGCATTACTTAGCTTATG	
qRT-*CAN2*-R	GCCTATTATTCTTTCTCCAGTCC	
qRT-*EIN2*-F	TGCAGCAGCTAATGTGTTTCA	
qRT-*EIN2*-R	GCGGGTATTTCTATCTTCAGGA	qRT-PCR
qRT-*EIN3*-F	TCAAGGCTTTGTTTATGGGAT	
qRT-*EIN3*-R	GCAAGGTATGAGGAGTCGGT	
qRT-*AMS*-F	GATGGACGATTCTGTAACG	
qRT-*AMS*-R	AGGCAAGAAATTGAGTCG	
qRT-*DYT1*-F	ATTGTGTAAGATTGGGGAG	
qRT-*DYT1*-R	AACAGAGGCACTAATAAGAAT	
qRT-*TDF1*-F	CAGCTACTAGCGACCTCAC	
qRT-*TDF1*-R	GACAACATCTCTTGGTCTTTA	

qRT-*MS188*-F	GAAGAAGAAGTTGTCAGGAA
qRT-*MS188*-R	GTGAGCAAGTGAAGCATCT
qRT-*MS1*-F	ACTTAACCGGTCATCAGGTCATG
qRT-*MS1*-R	CGATATCCATCGGTATTCGACTC
qRT-*ERF109*-F	TGTTTGGCTTGGTACTTTCG
qRT-*ERF109*-R	GCACTTGCCTTTGCTCCTAT
qRT-*SAG2*-F	CAGATCTTAGGTCAATCTCGTC
qRT-*SAG2*-R	TGGTGGATCTGATCAAATCAAG
qRT-*SAG20*-F	TCCTTCGAATTCGTACCTCT
qRT-*SAG20*-R	AGTACTCCCTCACCTGTGTG
qRT-*ERF1*-F	CAGTCCACGCAACAAACCTA
qRT-*ERF1*-R	CCGAGCCAAACCCTAATACC
qRT-*ERF7*-F	TCTCACCTACCTCCACAATC
qRT-*ERF7*-R	CGGTCTCGGTCCGCTACAAG

Table S2. The transcripts of *ERF*s and *SAG*s were detected in the tapetum.

Name	ID	stage 6 and 7	stage 8 - 10
SAG20	AT3G10985.1	71	83
SAG2	AT5G60360.1	19	23
ERF109	AT4G34410.1	116	77
ERF7	AT3G20310.1	71	64
ERF3	AT1G50640.1	38	39

The data shows the detailed reads number extracted from the transcriptome of tapetal cells (at stage 6-7 and stages 8-10) captured by laser microdissection and pressure catapulting (LMPC) [39].
